# Timing of chemotherapy-induced neutropenia predicts prognosis in metastatic colon cancer patients: a retrospective study in mFOLFOX6 -treated patients

**DOI:** 10.1186/s12885-017-3240-6

**Published:** 2017-04-04

**Authors:** Yang Chen, YanRong Wang, Yan Shi, GuangHai Dai

**Affiliations:** Medical Oncology Department 2, Chinese People’s Liberation Army General Hospital, Beijing, 100853 People’s Republic of China

**Keywords:** Metastatic colon cancer, Timing of chemotherapy-induced neutropenia (CIN), Prognosis, Chemotherapy

## Abstract

**Background:**

The occurrence of Chemotherapy-induced neutropenia (CIN) was reported to be a predictor of better survival in several cancers. The objective of our study is to evaluate the relationship between the timing of CIN and prognosis.

**Methods:**

Between June 2012 and August 2014, 290 patients with confirmed metastatic colon cancer received at least one cycle of mFOLFOX6 as first-line chemotherapy were eligible for assessment as all patients group. Of the 232 received at least six cycles of mFOLFOX6 and survived 150 days after treatment were considered as landmark group. Timing of CIN was categorized into absence, early-onset and late-onset CIN groups. The end of cycle 3 was the cutoff to differentiate early-onset or late-onset. The correlation between timing of CIN with survival was analyzed by Kaplan-Meier method and Cox proportional hazards model.

**Results:**

In all patients group, the median survival of patients without neutropenia, early-onset and late-onset neutropenia were 6.7, 20.7 and 12.8 months (*P* < 0.001). The patients with early-onset and late-onset CIN had better prognosis than CIN absence by multivariate analysis. Findings were much the same for landmark group.

**Conclusions:**

In conclusion, timing of CIN is an independent predictor of prognosis in metastatic colon cancer patients received mFOLFOX6, whereas an early-onset of CIN predicts longer survival.

## Background

Colon cancer is the third most commonly diagnosed cancer and the third leading cause of cancer mortality in both men and women in United States [1]. Most patients diagnosed with metastatic colon cancer are offered chemotherapy. Although targeted drugs, such as epidermal growth factor receptor antibody (cetuximab and panitumumab), are recommended as an optional component of first-line treatment for genetically susceptible tumors, oxaliplatin combined with 5-fluorouracil or its derivatives doublet chemotherapy is the standard and most commonly used treatment in metastatic colon cancer patients. Hence, there is a great need to identify patients who are more likely to benefit from chemotherapy.

Chemotherapy-induced neutropenia (CIN) is one of the most common adverse effects which often lead to dose reduction even withdrawal from treatment. Since the late 1990s, several studies reported the association of CIN with a better clinical outcome in breast cancer patients [2–4]. The similar association was also found in gastric cancer [5], pancreatic cancer [6], non-small cell lung cancer (NSCLC) [7], small-cell lung cancer [8], metastatic and refractory colorectal cancer (CRC) [9–11]. In addition, Jang et al. [12] raised a new viewpoint that timing of CIN might be a predictive factor for favorable prognosis in patients with NSCLC. Whether the timing of CIN or severity of CIN is the main prognostic factor remains unclear. The objective of this study is to investigate the clinical implication of CIN and the possible correlation between timing of CIN with prognosis in metastatic colon cancer patients.

## Methods

### Patients and data collection

This was a retrospective study approved by the ethics committee of Chinese People’s Liberation Army (PLA) General Hospital. From June 1, 2012 to August 31, 2014, patients with metastatic colon cancer admitted for chemotherapy were included for analysis. Before chemotherapy, written informed consent was submitted from the patients or their legal guardian. All blood tests and treatments were performed in accordance with institutional guidelines. Clinical data were retrieved from the medical records of PLA General Hospital database.

**Fig. 1:**
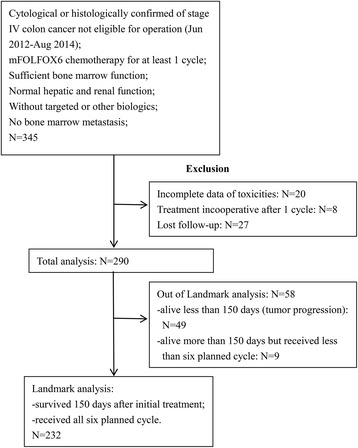
Study flow chart

The inclusion criteria were: 1) patients were cytological or histologically confirmed stage IV colon cancer and not eligible for operation; 2) patients received at least one cycle of mFOLFOX6 as first-line treatment; 3) sufficient bone marrow function; 4) normal hepatic and renal function; 5) without targeted or other biologics; 6) no bone marrow metastasis. Exclusion criteria: 1) incomplete data of toxicities; 2) lost follow-up; 3) treatment incooperative after 1 cycle. Total 290 patients with confirmed metastatic colon cancer patients received at least one cycle of mFOLFOX6 as first-line chemotherapy were eligible. To avoid selection bias due to a higher chance of neutropenia with increasing cycles of chemotherapy as a result of an inherently better prognosis, we used a cutoff time of 150 days after initial treatment to restrict primary analyses to 232 patients who received all six planned cycles of chemotherapy, and survived 150 days after initial treatment, which was defined as the landmark group [7, 13]. Since landmark analysis has been widely used in clinical investigations for adverse events assessment in chemotherapy, we investigated the clinical implication of timing of CIN in both all patients group and landmark patients group. We followed up until May 31, 2016 to obtain clinical information. Specific details of enrollment and exclusion were also showed in the following flow chart (Fig. 1).

### Dose intensity of chemotherapy

To avoid bias caused by different chemotherapy drugs, patients in this study all received mFOLFOX6 chemotherapy, which is a 2-week treatment per cycle. Patients underwent at least one cycle of mFOLFOX6 as first-line chemotherapy, which consisted of 5-Fu (400 mg/m^2^ IV bolus on d1, then 2400 mg/m^2^ IV continuous infusion over 46 h), oxaliplatin (85 mg/m^2^ IV, d1) and leucovorin (400 mg/m^2^ IV, d1). The relative dose intensity (RDI) was recorded as the ratio of delivered dose intensity of chemotherapy to standard dose intensity within 6 cycles. A dose reduction was required with grade 3 or higher hematological toxicity based on our institutional treatment protocol.

### Assessment of neutropenia

Blood samples were routinely taken prior to chemotherapy (Day 0 or 1) and every 7 days. Absolute neutrophil count (ANC) was calculated by multiplying the white blood cell count by the total percentage of neutrophils. CIN absence was defined as ANC > 2.0 × 10^9^ / L. According to National Cancer Institute (NCI) Common Terminology Criteria for Adverse Events (CTCAE, version 4.0), CIN grade was defined as follows: Grade 1, ANC 1.5–2.0 × 10^9^ / L; Grade 2, ANC 1.0–1.5 × 10^9^ / L; Grade 3, ANC 0.5–1.0 × 10^9^ / L; and Grade 4, ANC 0–0.5 × 10^9^ / L. Grade 1 and Grade 2 neutropenia were considered as mild neutropenia and Grade 3 and Grade 4 neutropenia were considered as severe neutropenia. The patients with CIN were also categorized into early-onset (E) group and late-onset (L) group according to the time of CIN occurrence. Group E and Group L were defined as CIN presence before and after the end of cycle 3, respectively. Both the timing and severity of neutropenia for each patient were recorded for analysis.

### Evaluation of the chemotherapy efficacy and survival

Response Evaluation Criteria in Solid Tumors, version 1.1 (RECIST 1.1) was used for response evaluation for objective response rate (ORR) and disease control rate (DCR). Progression free survival (PFS) and overall survival (OS) were defined as the time from the date of patient’s initial treatment to date of tumor progression or date of patient’s death.

### Statistical analysis

The Wilcoxon and Pearson’s chi-square tests were used to determine difference among different groups. Survival curves were estimated by the Kaplan–Meier method and compared by the log-rank test. Multivariate survival analyses were performed using Cox proportional hazards regression models, which included KPS, pathological differentiation, liver metastasis, severity of CIN and timing of CIN. All of the analyses were performed with the statistical software packages R (http://www.r-project.org, The R Foundation) and EmpowerStats (http://www.empowerstats.com, X&Y Solutions, Inc., Boston, MA). All statistical tests were two-tailed and 0.05 was used to evaluate statistical significance.

## Results

### Demographics

Total 290 patients with histologically confirmed metastatic colon cancer who received at least one cycle of mFOLFOX6 as first-line chemotherapy were eligible for assessment as all patients group. Of the 232 patients who received at least six cycles of mFOLFOX6 and survived 150 days after treatment were considered as landmark group (Fig. 1). Table 1 showed clinical characteristics of all patients and landmark group patients.

**Table 1:** Clinical characteristics of patients by timing of CIN in all and landmark groups with metastatic colon cancer received mFOLFOX6 as first-line treatment

	All patients				Landmark patients			
	Early-onset	Late-onset	Absence	*P* value	Early-onset	Late-onset	Absence	*P* value
	*N* = 141	*N* = 40	*N* = 109		*N* = 130	*N* = 32	*N* = 70	
Age(years)								
Median(range)	57 (29–74)	57 (37–73)	58 (33–76)	0.560	58 (49–62)	57.5 (54–61.5)	57 (53–62)	0.326
Height(cm)								
Median(range)	168 (153–179)	170 (153–184)	168 (154–180)	0.624	168 (165–170)	170 (160–176)	169 (165–170)	0.684
Weight(kg)								
Median(range)	67 (47–85)	72 (53–97)	64 (50–88)	0.058	62 (58–70)	66 (64–69)	73 (68–80)	0.043
Relative dose intensity								
Median	0.86 (0.73–0.89)	0.88 (0.75–0.91)	0.90 (0.78–0.93)	0.106	0.85 (0.71–0.87)	0.89 (0.74–0.90)	0.88 (0.78–0.91)	0.215
Gender								
male	48	17	36	0.752	45	15	31	0.214
female	93	23	73		85	17	39	
KPS								
90	131	39	103	0.216	121	31	66	0.069
70–80	10	1	6		9	1	4	
Pathological differentiation								
Well-moderate	121	25	83	0.039	114	21	52	0.283
Poor	20	15	26		16	11	18	
Liver metastasis								
present	110	27	85	0.502	102	21	55	0.368
absent	31	13	24		28	11	15	
Severity of CIN								
Absence	0	0	109	<0.01	0	0	70	<0.01
Mild	107	25	0		98	22	0	
Sever	34	15	0		32	10	0	

Tests used: Wilcoxon and Pearson’s chi-square tests

### The feature of CIN

In all patients, 181 (63%) patients experienced neutropenia. Among them, 141 (78%) patients were early-onset CIN and 40 (22%) patients were late-onset CIN, while 132 (73%) patients were mild CIN and 49 (27%) were severe CIN. In landmark group patients, 162 (70%) patients experienced neutropenia. Among them, 130 (80%) patients were early-onset CIN and 32 (20%) patients were late-onset CIN, while 120 (74%) patients were mild CIN and 42 (26%) were severe CIN. There was no significant difference among groups by timing of CIN in age, gender, presence of liver metastases and KPS scores, as well as relative dose intensity (all *P* > 0.05) (Table 1).

### Survival analysis

**Fig. 2:**
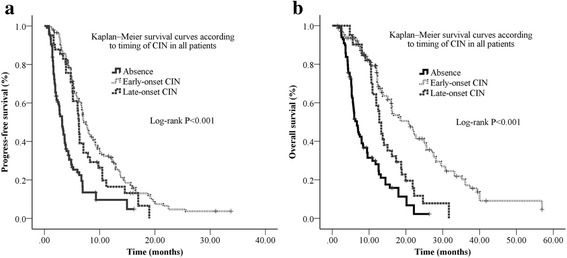
Kaplan–Meier survival curves according to timing of CIN in all patients. **a** The median PFS in the CIN absence group, early-onset CIN group, and late-onset CIN group were 3.2 months (95% CI: 2.7–3.8), 7.2 months (95% CI: 6.4–8.0), and 6.2 months (95% CI: 5.9–6.6), respectively. **b** The median survival in CIN absence group, early-onset CIN group, and late-onset CIN group were 6.7 months (95% CI: 5.6–7.8), 20.7 months (95% CI: 16.1–25.4), and 12.8 months (95% CI: 11.6–13.9), respectively

**Fig. 3:**
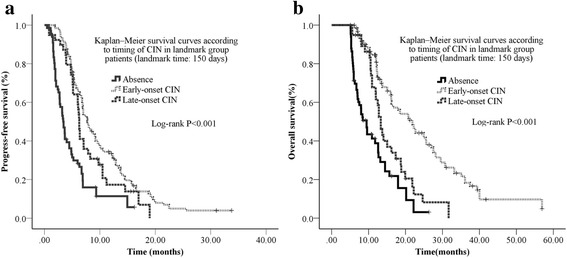
Kaplan–Meier survival curves according to timing of CIN in landmark group patients (landmark time: 150 days) **a** The median PFS in CIN absence group, early-onset CIN group, and late-onset CIN group were 3.4 months (95% CI: 2.7–4.1), 7.7 months (95% CI: 6.5–8.9), and 6.3 months (95% CI: 5.9–6.6), respectively. **b** The median survival in CIN absence group, early-onset CIN group, and late-onset CIN group were 9.5 months (95% CI: 7.9–10.9), 21.9 months (95% CI: 18.1–25.7), and 13.3 months (95% CI: 11.9–14.7), respectively

We subsequently evaluated prognostic significance of severity of CIN and timing of CIN using univariate Kaplan-Meier survival analysis and multivariate Cox regression analysis. By May 31, 2016, the median overall survival (mOS) and the median progression free survival (mPFS) for patients experiencing neutropenia were longer than patients without neutropenia in all patients (mOS: 16.3 m vs 6.7 m, *P* < 0.001; mPFS: 6.9 m vs 3.2 m, *P* < 0.001) and landmark group patients (mOS: 18.5 m vs 9.5 m, *P* < 0.001; mPFS: 7.1 m vs 3.4 m, *P* < 0.001). Univariate analysis showed that patients with early-onset CIN and late-onset CIN had a longer survival than those without CIN in both all and landmark groups. In all patients group analysis, the mOS of patients without neutropenia (A), patients with early-onset neutropenia (E), and patients with late-onset neutropenia (L) were 6.7 months, 20.7 months and 12.8 months, (A vs E, *P* < 0.001; A vs L, *P* < 0.001; E vs L, *P* < 0.001), respectively (Fig. 2) (Table 2). In landmark patients group analysis, the mOS of group A, group E, and group L patients were 9.5 months, 21.9 months and 13.3 months, and (A vs E, *P* < 0.001; A vs L, *P* = 0.009; E vs L, *P* < 0.001), respectively (Fig. 3 and Table 2). In addition, the patients with early-onset CIN had the longest mPFS in both all patients and landmark patients groups, which was consistent with the results of OS (Figs. 2, 3 and Table 2).

**Table 2:** Univariate analysis for the association between clinical characteristics and survival in all and landmark groups with metastatic colon cancer received mFOLFOX6 as first-line treatment

	All patients					Landmark patients				
	PFS			OS		PFS			OS	
	N (%)	Survival (months) Median (95%CI)	*P* value	Survival (months) Median (95%CI)	*P* value	N (%)	Survival (months) Median (95%CI)	*P* value	Survival (months) Median (95%CI)	*P* value
KPS										
90	273 (94)	5.9 (5.1–6.9)	0.287	17.5 (8.1–22.8)	0.371	218 (94)	6.3 (5.6–6.9)	0.154	17.8 (17.1–23.9)	0.071
70–80	17 (6)	2.7 (2.6–2.9)		15.4 (9.8–18.9)		14 (6)	2.8 (2.6–3.7)		15.6 (10.9–20.9)	
Pathological differentiation										
Well-moderate	229 (79)	5.7 (4.6–6.8)	0.053	12.6 (11.6–13.7)	0.060	187 (80)	6.3 (5.2–7.4)	0.785	13.7 (10.9–16.6)	0.040
poor	61 (21)	5.3 (2.7–7.9)		10.2 (5.9–14.7)		45 (20)	6.2 (4.8–7.7)		12.0 (8.1–15.9)	
Liver metastasis										
presence	222 (76)	4.9 (4.0–5.9)	0.116	12.1 (10.7–13.6)	0.282	178 (77)	5.9 (4.9–6.9)	0.195	12.0 (8.1–15.9)	0.132
absence	68 (24)	6.9 (6.3–7.5)		16.3 (10.5–21.4)		54 (23)	6.2 (4.8–7.7)		12.8 (11.4–14.1)	
Severity of CIN										
Mild	132 (46)	6.9 (5.9–7.8)	<0.001	16.3 (13.4–19.2)	<0.001	120 (52)	6.9 (6.1–7.9)	<0.001	18.1 (13.6–22.4)	<0.001
Severe	49 (17)	7.2 (5.7–8.7)		16.1 (7.3–25.1)		42 (18)	7.6 (6.0–7.9)		18.8 (11.0–26.7)	
Absence	109 (37)	3.2 (2.6–3.8)		6.7 (5.4–7.9)		70 (30)	3.4 (2.7–4.1)		9.5 (8.0–10.9)	
M vs. A			<0.001		<0.001			<0.001		<0.001
L vs. A			<0.001		<0.001			<0.001		<0.001
M vs. L			0.416		0.139			0.360		0.127
Timing of CIN										
Early-onset	141 (49)	7.2 (6.4–8.0)	<0.001	20.7 (16.1–25.4)	<0.001	130 (56)	7.7 (6.5–8.9)	<0.001	21.9 (18.5–25.7)	<0.001
Late-onset	40 (14)	6.2 (5.9–6.6)		12.8 (11.6–13.9)		32 (14)	6.3 (5.9–6.6)		13.3 (11.9–14.7)	
Absence	109 (37)	3.2 (2.7–3.8)		6.7 (5.6–7.8)		70 (30)	3.4 (2.7–4.1)		9.5 (7.9–10.9)	
E vs. A			<0.001		<0.001			<0.001		<0.001
L vs. A			<0.001		<0.001			0.003		0.009
E vs. L			0.115		<0.001			0.095		<0.001

*Abbreviation CIN* chemotherapy-induced neutropenia, *M* mild CIN, *S* severe CIN, *A* absence of CIN, *E* early-onset CIN, *L* late-onset CIN

A two-sided significance level of 0.05 was used to evaluate statistical significance

Table 3 showed that the patients in group E (OS: HR = 0.378, 95% CI: 0.113–0.726, *P* = 0.021; PFS: HR = 0.501, 95% CI: 0.152–0.854, *P* = 0.032) and group L (OS: HR = 0.762, 95% CI: 0.219–0.865, *P* = 0.042; PFS: HR = 0.656, 95% CI: 0.275–0.932, *P* = 0.046) had better prognosis (OS and PFS) than group A by multivariate analysis in all patients cohort, which was in consistent with the results from the landmark group. However, we did not find any significant association between severity of CIN and clinical outcome by multivariate analysis in either all patients or the landmark group patients. Our data suggested that timing of CIN was an independent prognostic factor instead of severity of CIN for metastatic colon cancer patients who received mFOLFOX6 as the first-line treatment. According to multivariate analysis, pathological differentiation and KPS were also prognostic factors.

**Table 3:** Multivariate analysis for the association between clinical characteristics and survival in all and landmark groups with metastatic colon cancer received mFOLFOX6 as first-line treatment

	All patients				Landmark patients			
	PFS		OS		PFS		OS	
	HR (95%CI)	*P* value	HR (95%CI)	*P* value	HR (95%CI)	*P* value	HR(95%CI)	*P* value
KPS								
80 vs. 90^*^	0.963 (0.927–1.000)	0.052	1.039 (1.009–1.146)	0.041	1.077 (1.004–1.122)	0.013	1.016 (1.005–1.045)	0.046
Liver metastasis								
Presence vs. absence^﻿*^﻿	1.746 (1.127–2.706)	0.013	1.527 (0.963–2.422)	0.072	1.480 (0.967–2.265)	0.071	1.504 (0.911–2.482)	0.111
Pathological differentiation								
Well-moderate vs. poor^*^	1.006 (0.742–1.364)	0.096	0.658 (0.472–0.918)	0.014	1.050 (0.757–1.456)	0.770	0.626 (0.434–0.904)	0.012
Timing of CIN								
Early-onset vs. Absence^*^	0.501 (0.152–0.854)	0.032	0.378 (0.113–0.726)	0.021	0.698 (0.161–0.937)	0.042	0.471 (0.109–0.834)	0.023
Late-onset vs. Absence^*^	0.656 (0.275–0.932)	0.046	0.762 (0.219–0.865)	0.042	0.776 (0.303–0.947)	0.032	0.828 (0.230–0.957)	0.041
Severity of CIN								
Mild CIN vs. Absence^*^	0.580 (0.166–2.033)	0.395	0.509 (0.145–1.787)	0.292	0.437 (0.095–2.015)	0.288	0.433 (0.095–1.976)	0.280
Severe CIN vs. Absence^*^	0.525 (0.164–1.681)	0.278	0.606 (0.185–1.982)	0.407	0.383 (0.090–1.619)	0.192	0.487 (0.115–2.064)	0.329

^*^The later after vs. was the reference. Hazard ratios of survival with 95% CI were estimated with Cox’s proportional hazards model

Adjusted for: KPS; Pathological differentiation; Liver metastasis; Severity of CIN; Timing of CIN

### Efficacy analysis by timing of CIN

We also evaluated the correlation between timing of CIN and treatment response within 6 cycles. In all patients group, the objective response rates (ORRs) of group E and group L were significantly higher than that of CIN-absence group (55.3% vs 45.5% vs 17.6%, *P* < 0.001), while the disease control rates (DCRs) of group E and group L were significantly higher than that of CIN-absence group (88.2% vs 81.8% vs 50%, *P* < 0.001). The ORR and DCR were consistently higher in group E and L than group A in either all patients group or the landmark patients group (*P* < 0.001) (Table 4).

**Table 4:** Efficacy analysis by timing of CIN during first-line chemotherapy

	All patients				Landmark patients			
Timing of CIN	ORR	*P* value	DCR	*P* value	ORR	*P* value	DCR	*P* value
Early-onset CIN (%)	55.3 (78/141)	<0.001	87.9 (124/141)	<0.001	59.2 (77/130)	<0.001	92.3 (120/130)	<0.001
Late-onset CIN (%)	45.0 (18/40)		82.5 (33/40)		46.9 (15/32)		84.4 (27/32)	
Absence (%)	17.4 (19/109)		49.5 (54/109)		18.6 (13/70)		54.3 (38/70)	

## Discussion

Previous studies have shown the association between occurrence of CIN and better prognosis. Our results were in consistent with previous studies in metastatic colorectal cancer [9–11], the median OS, PFS of the patients experiencing neutropenia were longer than patients without neutropenia in both the whole patients (OS: 16.3 m vs 6.7 m, *P* < 0.001; PFS: 6.9 m vs 3.2 m, *P* < 0.001) and the landmark patients (OS: 18.5 m vs 9.5 m, *P* < 0.001; PFS: 7.1 m vs 3.4 m, *P* < 0.001). However, Martin Smoragiewicz et al. [14] did not find this association between CIN and clinical outcome for resected colon cancer. This suggested different immune status, tumor burden, or sensitivity to chemotherapy between metastatic colorectal cancer and resected colon cancer, which may affect the prognostic value of CIN. Therefore, we believe that the occurrence of CIN during chemotherapy is associated with favorable survival in metastatic colon cancer patients.

In addition, whether the timing of CIN or severity of CIN is a better prognostic factor was unclear in previous studies. Several investigations represented both occurrence and severity of CIN were well correlated with improved survivals in various cancers [5, 6, 8, 10, 15–20]. Only few studies investigated the correlation between timing of CIN and prognosis. Our study showed that early-onset CIN group and late-onset group had significantly better OS and PFS than CIN-absence group in both all and landmark patients groups by univariate and multivariate analysis, which was partially in consistent with the findings of Jang’s study. Jang et al. [12] found that although early-onset neutropenia group (within 2 cycles) showed significantly better PFS and OS than the late-onset group (3 to 6 cycles), there was no difference between the outcome of patients with late-onset and that of CIN absence group in 123 patients with advanced NSCLC. However, our study showed that even late-onset CIN group was better than CIN-absence group, which may due to difference in sample size, chemotherapy regimens and cut-off time of early or late-onset CIN. Our study was the first one to investigate the timing of CIN as a predictor for prognosis in metastatic colon cancer patients received mFOLFOX6 regimen.

CIN reflects the dose and pharmacokinetics of chemotherapy regimen, as well as patient’s genetic predisposition. In practice, the dose calculation for chemotherapy regimens has been based on patient’s estimated body surface area (BSA) [21]. However, literatures indicated this method did not account for the complex procedures of cytotoxic drug metabolism, distribution, and elimination [22]. Gurney demonstrated that there was a 4–10 fold variation in cytotoxic drug clearance between individuals. This can lead to an under-dosing of nearly 30% patients who received standard regimens [23]. Some investigators found it was effective to make dose escalation based on pharmacokinetics of the corresponding regimen. In a multiple center randomized study by Gamelin et al., a significantly improved objective response rate, trend of higher survival rate, and fewer grade 3/4 toxicities were observed in patients using individual fluorouracil (FU) dose adjustment based on pharmacokinetic monitoring [24]. Capitain et al. reported similar findings that the efficacy and tolerability of pharmacokinetically-adjusted FOLFOX dosing was much higher than traditional BSA dosing [25]. We believe using pharmacokinetically-adjusted chemotherapy is ideal for the treatment of colorectal cancer. However, in practice, it is not realistic to adjust the chemotherapy dosing for individual patient due to complex measure, especially in places with insufficient staff. Based on our findings, CIN is a potential surrogate marker of pharmacokinetics changes, and potentially a useful reference for physicians to adjust treatment dose.

As indicated in the editorial comments by Kvinnslan, the sensitivity of tumor cells to a chemotherapy regimen may reflect the patient’s genetic predisposition, and theoretically all cells in one patient sharing similar pharmacokinetics features of the regimen [26]. In another word, we think the tumor cell and neutrophil in a patient share a similar sensitivity to the treatment of a chemotherapy regimen. Our results showed that early-onset CIN indicated a better treatment response, PFS and survival in metastatic colon cancer patients, which suggested that patients with early-onset neutropenia might be the sensitive population to the mFOLFOX6 regimen. On the other hand, intrinsic and acquired drug resistance determines the efficiency of cancer chemotherapy [27]. The patients without neutropenia within 6 cycles in our study might be resistant to mFOLFOX6 regimen intrinsically, and even insensitive to other cytotoxic regimens result in unfavorable outcome. There were increasing investigations indicated genomic alterations including KRAS, BRAF, NRAS, and PIK3CA mutations [28], mismatch repair (MMR) status [29] and SMAD4 levels [30] were associated with the responses of chemotherapy, as well as clinical outcome. Unfortunately, our retrospective study did not have sufficient data on KRAS, BRAF, NRAS status, CEA levels, which was our limitation. It would be more meaningful to investigate the relationship among tumor genomic alterations, CIN and prognosis in metastatic CRC.

In addition, there was no significant difference in the median of relative dose intensity among three groups by timing of CIN in all patients and landmark group patients. It suggests that high-dose treatment or severe CIN induced by high-dose treatment is not necessarily advantageous for survival. However, absence of CIN could be considered as an indicator for insufficient dose of drug, insensitive to the regimen or unfavorable outcomes based on findings. Therefore, oncologists could potentially make a dose escalation or reduction from the recommend dosage according to CIN in addition to KPS and other toxicities.

## Conclusions

Our study suggested that the occurrence of CIN, rather than severity of CIN, might be a favorable indicator for efficacy of mFOLFOX6 and prognosis in metastatic colon cancer patients received mFOLFOX6 as first-line chemotherapy. We provide a potential surrogate biomarker, using timing of CIN to predict patient’s response to chemotherapy earlier, and further help with drug dosage adjustment. Further prospective investigation focusing on the comparison of clinical outcomes using fixed dosage versus dosage adjustment by CIN is warranted to validate our findings.

## Abbreviations

A: Absent
CIN: Chemotherapy-induced neutropenia
DCR: Disease control rate
E: Early-onset
L: Late-onset
ORR: Objective response rate
OS: Overall survival
PFS: Progression free survival
